# Numerical and experimental investigation of heat transfer enhancement in double tube heat exchanger using nail rod inserts

**DOI:** 10.1038/s41598-024-59085-5

**Published:** 2024-04-26

**Authors:** S. A. Marzouk, Fahad Awjah Almehmadi, Ahmad Aljabr, Maisa A. Sharaf

**Affiliations:** 1 Department of Mechanical Engineering, Faculty of Engineering, Kafrelsheikh University, Kafrelsheikh, 33511 Egypt; 2 Department of Applied Mechanical Engineering, College of Applied Engineering, Muzahimiyah Branch, King Saud University, P.O. Box 800, 11421 Riyadh, Saudi Arabia; 3 Department of Mechanical and Industrial Engineering, College of Engineering, Majmaah University, 11952 Al-Majmaah, Saudi Arabia; 4 Mechanical Engineering Dept, Faculty of Engineering, Damanhour University, Damanhour, 22511 Egypt

**Keywords:** Heat transfer enhancement, Nails rod insert, Exergy efficiency, CFD, Double tube heat exchanger, Mechanical engineering, Energy

## Abstract

The Double-tube heat exchanger (DTHX) is widely favored across various industries due to its compact size, low maintenance requirements, and ability to operate effectively in high-pressure applications. This study explores methods to enhance heat transfer within a DTHX using both experimental and numerical approaches, specifically by integrating a nail rod insert (NRI). A steel nails rod insert, 1000 mm in length, is introduced into the DTHX, which is subjected to turbulent flows characterized by Reynolds numbers ranging from 3200 to 5700. Three different pitches of NRI (100 mm, 50 mm, and 25 mm) are investigated. The results indicate a significant increase in the Nusselt (Nu) number upon the insertion of nail rods, with further improvements achievable by reducing the pitch length. Particularly noteworthy is the Nu number enhancement ratio for the 25 mm pitch NRI, which is 1.81–1.9 times higher than that for the plain tube. However, it is observed that pressure drop increases in all configurations with NRI due to heightened turbulence and obstruction by the NRI. Among the various pitch lengths, the 25 mm pitch exhibits the highest pressure drop values. Moreover, exergy efficiency is found to improve across all cases with NRI, corresponding to increased heat transfer, with the 25 mm pitch length showing a remarkable 128% improvement. Numerical analysis reveals that the novel insert enhances flow turbulence through the generation of secondary flows, thereby enhancing heat transfer within the DTHX. This study provides a comprehensive analysis, including temperature, velocity, and pressure drop distributions derived from numerical simulations.

## Introduction

A double-tube heat exchanger (DTHX) comprises two concentric tubes where one fluid flows inside the inner tube while another fluid circulates in the annular space between the inner and outer tubes, facilitating efficient heat transfer. This design is common in various industrial applications due to its simplicity, reliability, and effectiveness in thermal exchange processes^1^. Augmenting heat transfer in HXs could be done by various techniques such as using different configurations^2,3^, installing baffles^4–11^, fins^12^, vortex generators^13,14^, or adding nanofluids^15–17^. Common techniques applied to enhance the heat transferred in a DTHX are inserts, fins, nanofluids, and turbulators^18–22^. However, enhancing the thermal performance of DTHXs with inserts increases the pressure drop (Δp). Therefore, optimization was needed to carefully achieve the best design of DTHXs with inserts. Research regarding TTIs in a circular tube was done by Saha et al.^23^. They established that the spaced TTI in a circular tube had better performance than regular full-length TTI. An experimental work was prepared on the heat transfer and Δp of a circular tube linked to helical screw tapes by Sivashanmugam and Suresh^24^. A coiled wire was experimentally examined by Gunes et al.^25^ at different coil characteristics and Res. The insert of the coiled wire increased both heat transfer rate and Δp and concluded that the best application of it was at a low *Re*. Promvonge^26^ used coiled wire and twisted tape inside a tube and examined its effect over a range of Res. The outcomes established that heat rate augmentation with coiled wire and TTI was two times of only coiled wire or twisted tape at a low *Re*. Eiamsa-ard et al.^27^ used twin counter and twisted tapes besides the regular single twisted tape. The growth in the twist ratio decreased the *Nu*, thermal enhancement index, and Δp. Few empirical correlations were acquired in this research. The consequence of a solid hollow circular disk turbulator inserted in a tube was investigated experimentally by Kumar et al.^28^ at different geometrical parameters and Res. The results discovered that this insert augmented the thermal performance by 140%. Bhuiya^29^ experimentally analyzed the influences of using perforated TTIs in a circular tube over a range of *Re* at four different porosities. The porosity of 4.5% was found to be the optimum among the four cases of different porosities. Another experimental work was applied by Salam et al.^30^ on a PT having rectangular cut TTI. The heat transfer and the Δp increased by about 200% and 160%, respectively. A three-dimensional model of helical baffles in a DTHX was conducted by El Maakoul et al.^31^. The model was performed at various baffle spacing and Res and presented that heat transfer and Δp augmented with the growth of baffle spacing and *Re*. Prasad et al.^32^ experimentally examined the helical tape inserts in the tube side with nanofluid flow. The outcomes presented that the nanofluid and helical tape inserts improved heat rate through a rise in Δp. The same result by utilizing helical tape inserts on the tube side was reported^33^. Sadeghi et al.^34^ studied using different nanofluids in a DTHX through a helical tape insert. They found that the nanofluids resulted in higher *Nu* than using water. Some studies that examined different properties of TTIs in a DTHX were introduced. Chaurasia and Sarviya^35^ compared one and paired strip helical-screw tape inserts using nanofluid. HX size decreased by using this type of insert. Hangi et al.^36^ performed a parametric study looking for an optimum case in a DTHX with four types of helical strips on the annulus side and a helical insert on the tube side where nanofluid flows. It was found that when the nanoparticles are homogeneously distributed in the cases with higher mixing, the heat transfer enhancement was augmented. Najafabadi et al.^37^ suggested shapes that raised the effective contact area at a constant volume to enhance the heat rate between water in the inner pipe and the phase-change material on the annulus side. Several geometrical and fluid parameters were examined to find the optimum case. Shell and tube HX performance was experimentally evaluated by^38^. They utilized circular rod inserts prepared by wire nails to study the air injection impact on shell-tube HX performance. The results revealed that there was a noteworthy development of heat transfer by insertion than that of air injection. An experimental investigation by Murugesan^39^ was presented to study Δp and *Nu* of DTHX using wire nails in twisted tape. The thermal enhancement factor, Δp, and *Nu* with higher values are obtained with the insertion compared with the PT. Kumar et al.^40^ experimentally examined the extent strip inserts inside a DTHX where with Fe_3_O_4_ nanofluid. Different Fe_3_O_4_ nanofluid concentrations were used at various *Re* and various strip aspect ratios to study impacts on Δp, heat transfer rate, and effectiveness. The *Nu* was proportional to *Re* and nanofluid concentration, but disproportional to the strip aspect ratio. Bas and Ozceyhan^41^ experimentally examined the use of TTIs inside a tube where the inserts are separated from the tube's inner wall by Teflon rings at contact points. Splitting the inserts from the inner wall was to prevent any contamination at the contact points. Different clearance ratios and twist ratios were studied at various *Re* and various strip aspect ratios to study their effects on Δp, and heat transfer rate. Kumar et al.^42^ explored the impact of circular perforated rings within a circular tube. Different thicknesses, diameters, pitch ratios, and perforation index were studied at a range of *Re* to examine their influences on Δp and heat transfer. Virgilio et al.^43^ examined the turbulent-flow in pipes with continuous and discontinuous helicoidal turbulators at different pitch-to-diameter-ratio. They found that the discontinuous helicoidal turbulators have lower pressure losses and turbulent kinetic energy. Moya-Rico et al.^44^ studied the impact of DTHX on thermo-hydraulic performance using a water/sugar solution with smooth and corrugated tubes. The results highlighted an increment of 19% compared to the smooth tube.

The literature review reveals that TTI, fins, and spring coils have been employed in several HXs to improve heat transfer however NRI was not used as an insert in DTHX. Additionally, NRI offers many benefits over other inserts, including quick production, low cost, simple insertion and removal, and reduced foaling. It was discovered, as reported in the literature mentioned above, that vortex generators were more effective than centrifugal forces for enhancing heat transfer in situations when NRI is predicted to do so. In this study, the effects of a NRI with different pitches to augment the heat transfer in DTHX will be analyzed experimentally and numerically. This innovative insert, distinct from conventional solutions, is designed to augment heat transfer significantly. The effects of different pitches of NRI such as 100 mm, 50 mm, and 25 mm will be investigated. The parameters of *Nu*, Exergy efficiency, and Δp will be used to evaluate the proposed model's effectiveness in DTHX.

## Experimental study

### Experimental setup

**Figure 1:**
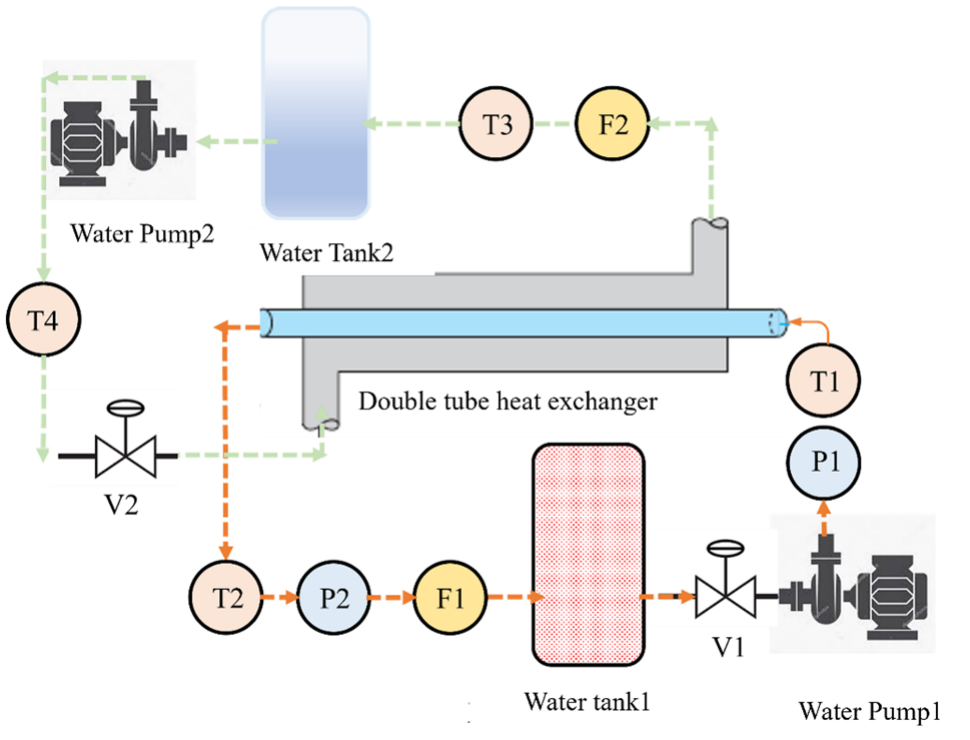
A schematic view of the experimental study.

A schematic view of the investigational setup is illustrated in Fig. 1 where there are two loops (hot-water loop, and cold-water loop). The hot fluid moves in the internal copper tube with a 19 mm diameter and the cold water moves in the external steel tube with a 50 mm diameter. An electrical heater heats the water in water tank 1 with a 4 KW capacity then it is driven by centrifugal water pump 1 to pass through the internal tube. To control the fluid flow, two control valves (V1 and V2) are utilized. Flow, pressure, and temperature sensors are used to record the results during experiments. The cold water is chilled in water tank 2 by city water where water pump 2 forces cold water to the external tube of the DTHX. The annulus side flow rate is kept constant whereas the flow rate at the internal tube is variable. The inlet tube temperature was kept at 70 °C, while the inlet tube temperature at the internal side was kept at 30 °C.

### Instrumentation devices

The internal and external tube's inlet and exit water temperatures were determined using four K-thermocouples (T1–T4) placed within the flow path to measure the temperature values. An ARDUINO circuit was employed for data recording with a personal computer. A flow rate sensor with an accuracy of 2.3% was used to monitor the hot and cold-water flow rates. The pressure drop across the internal tube was determined using pressure sensors (P1 and P2) positioned at the inlet and outlet of the tube. The flow rates of hot and cold water are determined by flow sensors F1 and F2, respectively. Further details about the sensors utilized in the experiments are provided in Table 1.

**Table 1:** Types and specifications of sensors.

Type	Description	Accuracy
K-type thermocouple	Temperature	± 1.1%
YF-S201	Flow rate	± 2.3%
G1/4 inch	Pressure	± 1.7%

### The configurations of insert

**Figure 2:**
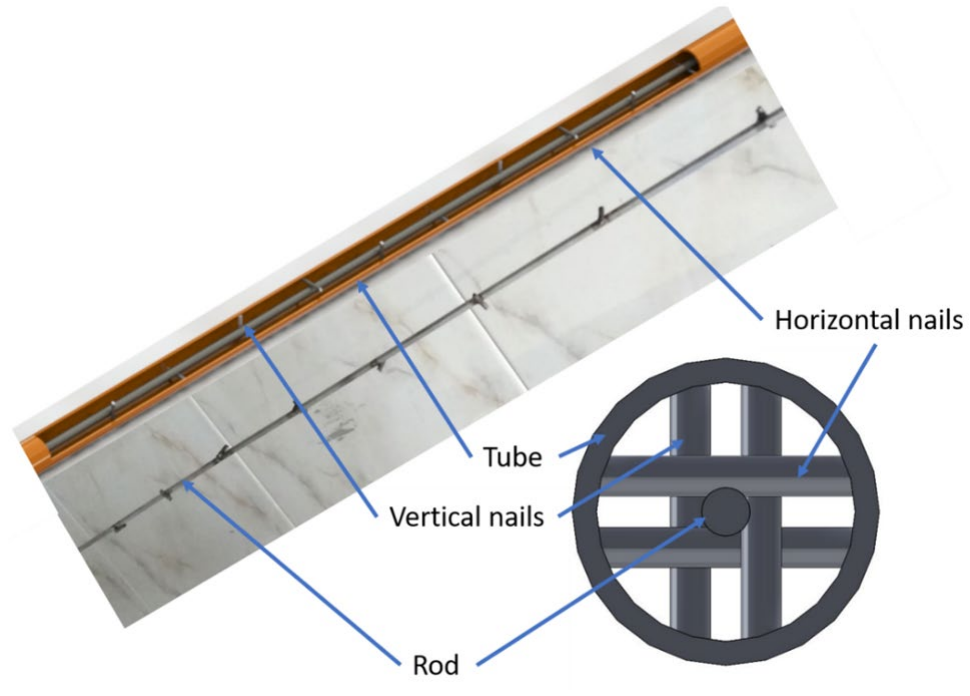
Photograph and schematic view of nails rod inserts (NRI) inside copper tube.

**Figure 3:**
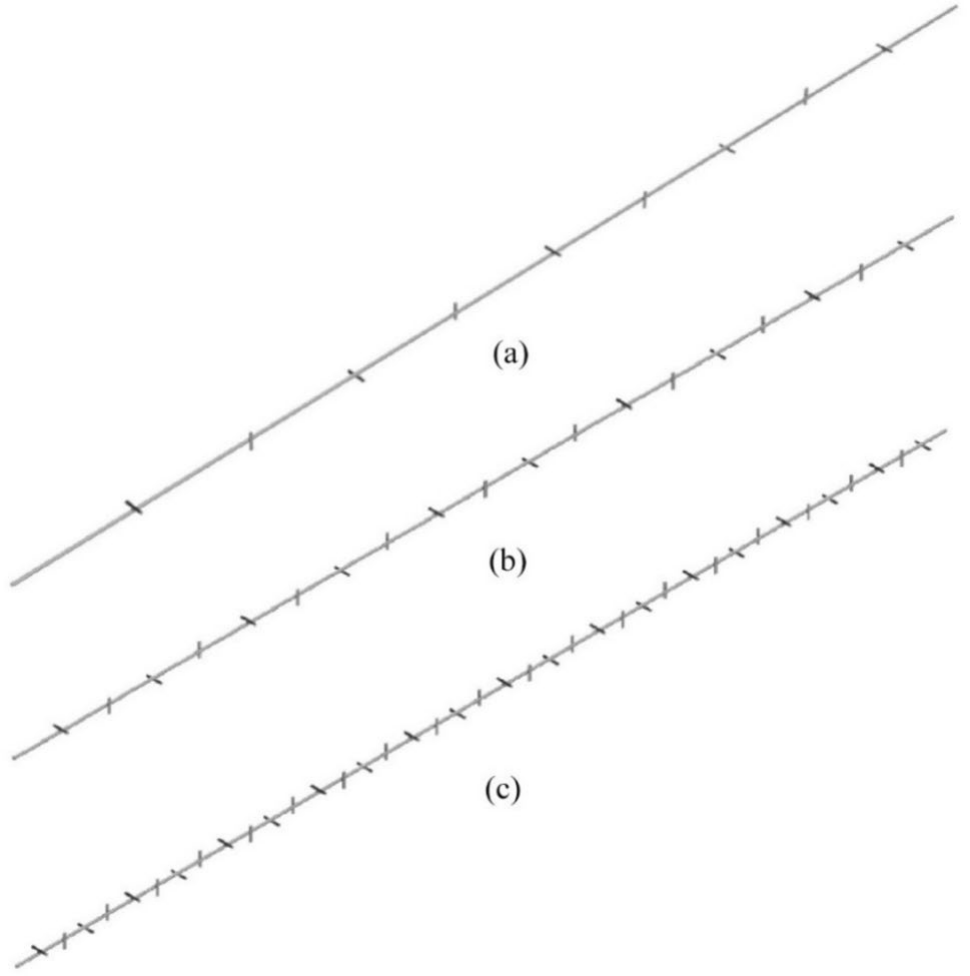
The NRI configurations with different pitches (**a**) 100 mm, (**b**) 50 mm, (**c**) 25 mm.

Figure 2 represents the schematic view of NRI. The rod used is made of steel of 1000 mm in length, and 3 mm in diameter, and equipped with wire nails. Nails that are 17 mm in length and 2.5 mm in diameter with different pitch lengths are used. The nails are arranged alternately vertically and horizontally on the rod surface. The NRI configurations with different pitches such as 100 mm, 50 mm, and 25 mm are investigated in Fig. 3. The details of the novel nail rod insert (NRI) used in the study are illustrated in Table 2.

**Table 2:** Details of NRI inserts.

Name	Pitch (mm)	Number of nails
Configuration 1	100	9
Configuration 2	50	19
Configuration 3	25	39

### Data reduction

The transferred heat by the cold water on the annulus side and hot water in the inner tube are calculated from ${Q}_{as}={\dot{M}}_{cw}{c}_{p.cw}\left({T}_{cw.out}-{T}_{cw.in}\right)$ and ${Q}_{int}={\dot{M}}_{hw}{c}_{p.hw}\left({T}_{hw.in}-{T}_{hw.out}\right)$, respectively. The average heat is calculated using^45,46^.

1 $${Q}_{ave}=\frac{{Q}_{as}+{Q}_{int}}{2}$$

The heat balance deviation $\frac{{Q}_{as}+{Q}_{it}}{{Q}_{ave}}$ has to be lower than 5%. The U is calculated from^47^:

2 $$U=\frac{{\dot{Q}}_{Ave}}{{A}_{s}F\Delta {T}_{m}}$$

where *F* is the correlation factor and the surface area of the inner tube (${A}_{s}$) is $n\pi DL$, where n is the number of tubes which equals one and the logarithmic mean temperature difference is estimated by^48^:

3 $$\Delta {T}_{Lm}=\frac{\left({T}_{hw.out}-{T}_{Cw.out}\right) -\left({T}_{hw.in}-{T}_{Cw.in}\right)}{ln\frac{\left({T}_{hw.out}-{T}_{Cw.out}\right)}{\left({T}_{hw.in}-{T}_{Cw.in}\right)}}$$

The friction factor is crucial for assessing the pressure drop during fluid flow. It directly influences the energy requirements, pump sizing, and overall system efficiency which can be calculated by^49^:

4 $$f=\frac{\Delta p}{\rho \frac{l}{{D}_{h}}\frac{{V}^{2}}{2}}$$

The *Nu* in a DTHX is a dimensionless parameter used to characterize the convective heat transfer between the fluid stream inside the tubes and the annulus side. The *Nu* can be calculated by^50–52^:

5 $${{\text{N}}}_{{\text{u}}}=\frac{\mathrm{h }{{\text{D}}}_{{\text{h}}}}{{\text{K}}}$$

The exergy efficiency (η_ex_) is calculated from^53^:

6 $${\upeta }_{{\text{ex}}}=1-\left\{\frac{{{\text{T}}}_{{\text{o}}}\left[{\dot{{\text{M}}}}_{{\text{H}}}{c}_{p.hw}{\text{ln}}\left(\frac{{T}_{hw.out}}{{T}_{hw.in}}\right)+{\dot{{\text{M}}}}_{{\text{C}}}{c}_{p.cw}{\text{ln}}\left(\frac{{T}_{cw.out}}{{T}_{cw.in}}\right)\right]}{{\dot{{\text{M}}}}_{{\text{H}}}{c}_{p.hw}\left[\left({T}_{hw.out}-{T}_{hw.in}\right)-{{\text{T}}}_{{\text{o}}}{\text{ln}}\left(\frac{{T}_{hw.out}}{{T}_{hw.in}}\right)\right]}\right\}$$

where T_o_ is the ambient temperature, which in this analysis had a value of 30.1 °C.

### Experimental uncertainty

Uncertainty analysis in a DTHX involves assessing the reliability of experimental measurements to enhance the accuracy of results. Key sources of uncertainty include instrument precision, sensor placement, calibration, and variability in fluid properties. Understanding the impact of each factor allows for more robust conclusions about the heat exchanger's performance. Transparent documentation of procedures and calibration efforts is vital for ensuring the credibility and reproducibility of experimental findings. Comprehensive uncertainty analysis plays a pivotal role in refining measurement techniques and advancing the understanding of DTHX behavior. The uncertainties of the measured variables (temperature, Δp, and flow rate) are expressed as:

7 $${{\text{U}}}_{x}=\sqrt{{{B}_{x}}^{2}+{{D}_{x}}^{2}}$$

While systematic uncertainty is computed in Bx and random uncertainty is denoted by Dx, the uncertainty associated with result variables relies on various significant factors and can be determined by:

8 $${{\text{U}}}_{R}=\sqrt{\left(\frac{\partial R}{\partial {X}_{1}} {U}_{{X}_{1}}\right)^{2}+\left(\frac{\partial R}{\partial {X}_{2}} {U}_{{X}_{2}}\right)^{2}+\cdots \cdots +\left(\frac{\partial R}{\partial {X}_{n}} {U}_{{X}_{n}}\right)^{2}}$$

This study involved estimating and documenting the experimental uncertainties associated with the primary variables, and the results are presented in Table 3.

**Table 3:** Uncertainties in experimental results for studied parameters.

Variables	Uncertainty [%]
Water flow rate	± 0.32
Temperature	± 0.39
Nu	± 2.14
Exergy efficiency	± 4.12
*∆p*	± 3.57

## Numerical study

### Physical model

**Figure 4:**
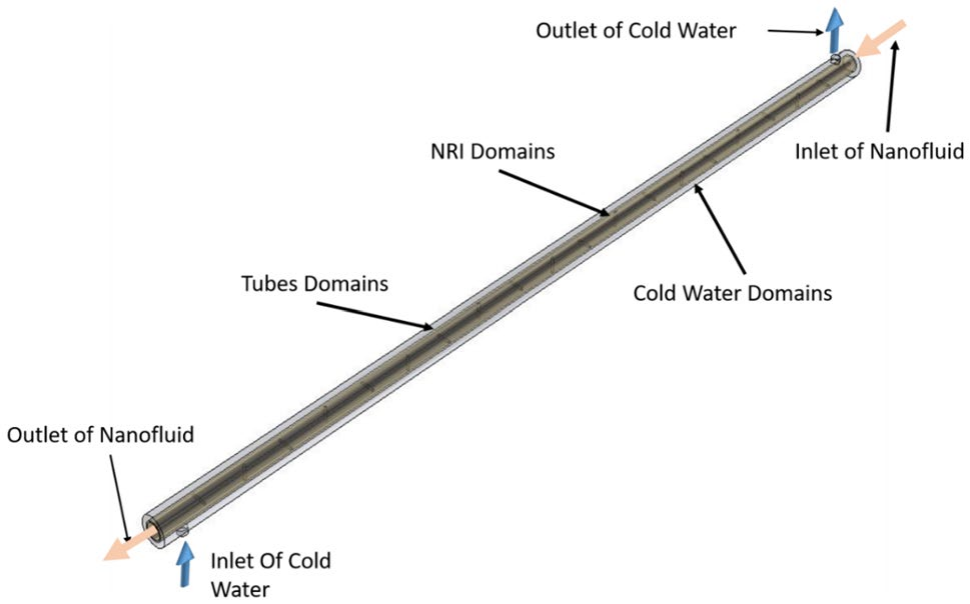
Numerical model of DTHX with NRI.

In the DTHX, the hot fluid passes through the hot tube transferring heat to the cold fluid and NRI is inserted inside the hot tube. Nails are placed in vertical and horizontal positions on the rod. The physical model of the numerical study consists of the hot and cold water fields, tube domains, and nail rods inserted in the hot water side. The physical model is completed in Solidworks software as depicted in Fig. 4 where the flow shape is in counter flow.

### Mesh generation

**Figure 5:**
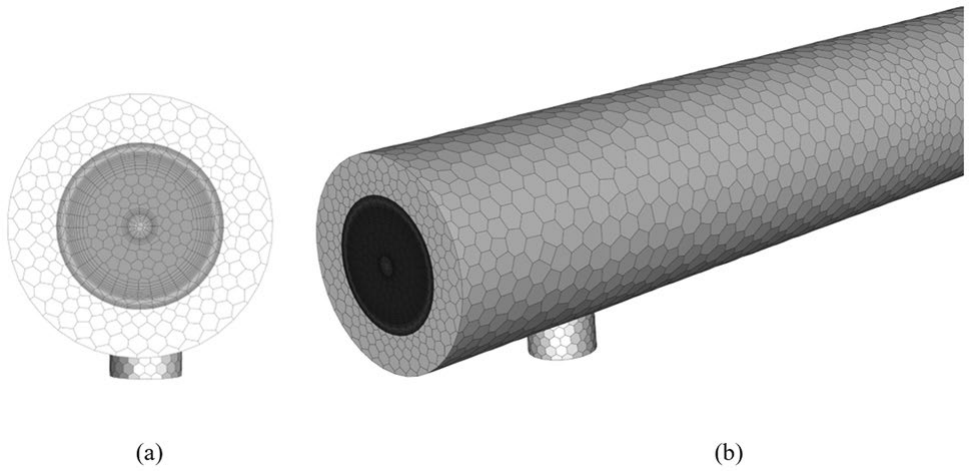
Mesh generation for (**a**) inflation layers at walls, (**b**) 3D models.

Selection of the precise mesh is necessary to obtain accurate results. The test section should be meshed in a small control volume. Figure 5 illustrates the mesh used in the present computational work which is generated by Ansys CFD software. The mesh is generated in a fine-structured hexahedral shape with a smaller element size near the NRI surface. Mesh inflation is used to enhance the boundary layer and monitor the turbulent flow effect near the wall.

The grid independence is checked for three various mesh numbers for example 123,562, 254,873, and 312,451 elements. The percentage of error in results reduces with the rising number of elements. The error for *Nu* is 3.55%, 2.46%, and 1.13% for 123,562, 254,873, and 312,451 elements systems, and those for pressure drop are 4.74%, 3.14%, and 1.38%, respectively. Accordingly, 312,451 cells were selected for the numerical model in this work.

### Boundary conditions and CFD procedure

The simulation study for the internal cold water, NRI, hot water, and copper tubes is conducted using Ansys Fluent. A pressure-based scheme is employed to achieve a steady state case after completing the three-dimensional model, which is then imported into Fluent. The realizable k-ɛ is chosen for the turbulent model, depending on prior research on different turbulence models for DTHXs through standard wall function^54^. Velocity inlets are set at the inlets of the internal side and annulus side, while pressure outlets are assigned at the outlets of the tube and the shell sides. Assumptions like incompressible fluids, steady fluid flow, constant properties, and no-slip walls are employed to simplify the simulation.

### Model validation

**Figure 6:**
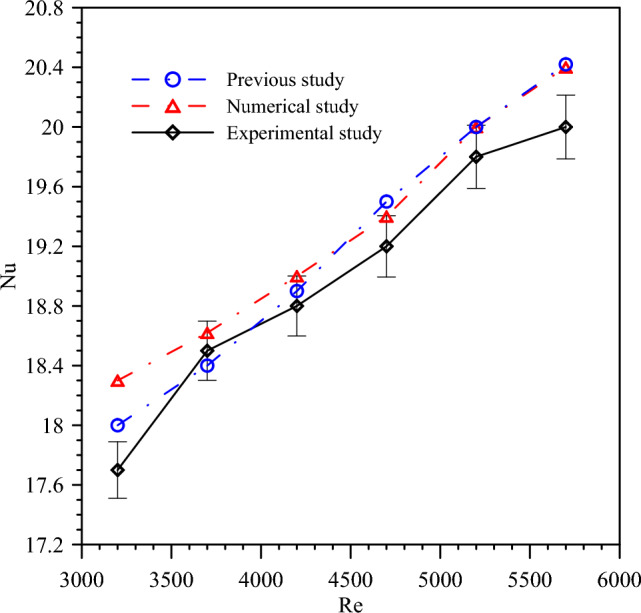
Comparing experimental results with numerical results for Nusselt number.

**Figure 7:**
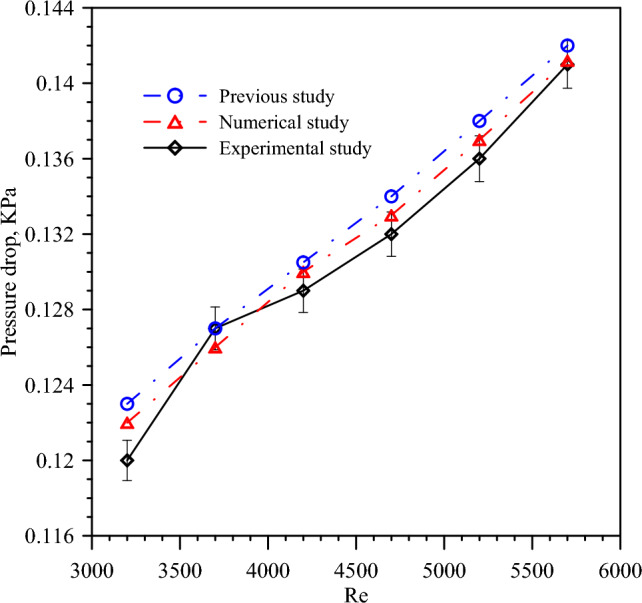
Variation of pressure drop with Re for experimental and numerical results.

The successful validation of the numerical model indicates that it accurately predicts the behavior of DTHX providing confidence in its predictive capabilities for scenarios. Figure 6 introduces a validation of the numerical model for PT and turbulent flow with *Re* number ranges from 3200 to 5700 with experimental results and previous results^55^. The deviation ranges for Nu number between experimental and numerical results are around 2.33% whereas the percentage error for pressure drop between experimental and numerical results are from 1.78% as seen in Fig. 7. The deviation ranges for Nu number between experimental and previous results are around 2.16% whereas the percentage error for pressure drop between experimental and previous results are from 1.52%. So, it is demonstrated that the numerical simulation model is effective and accurate. In general, the differences are reasonable, and the validation process ensures the accuracy and reliability of the numerical simulations.

## Results and conclusions

The influence of utilizing NRI in a DTHX is studied for heat transfer enhancement. *Nu*, Δp and exergy efficiency are calculated, and the numerical results are confirmed by comparing them with the experimental outcomes. Also, Temperature, velocity, and pressure distributions are presented.

### Heat transfer enhancement

**Figure 8:**
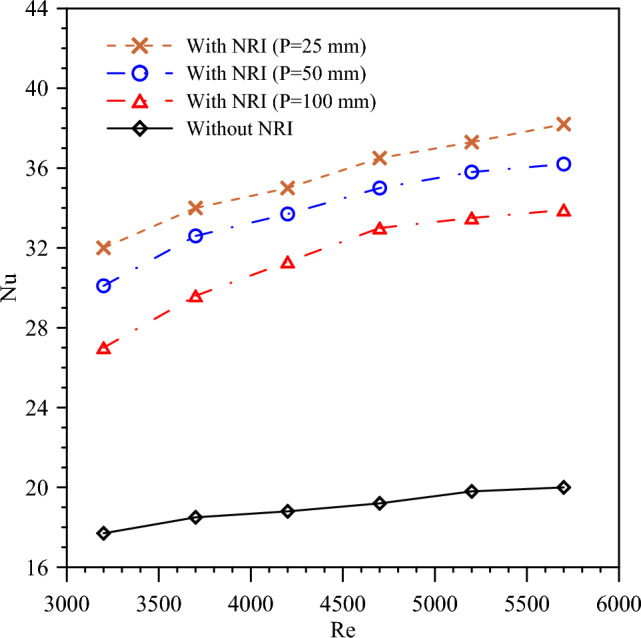
The variation of *Nu* versus *Re* for PT and NRI at various pitches.

Nusselt number is studied for turbulent flow with a PT and NRI at *Re* number ranges from 3200 to 5700. *Nu* augments with NRI that is because NRI increases swirl flow and reduces boundary layer thickness which increases the heat transfer as described in Fig. 8. The highest value of *Nu* is obtained at a pitch length of 25 mm and a *Re* number of 5700. Nus 19, 33, 35, and 36.5 are obtained by PT, NRI with 100 mm pitch, NRI with 50 mm pitch, and NRI with 25 mm pitch at a *Re* number of 4700, respectively. *Nu* increases by pith value decrease because low pitch values enhance the turbulent flow and as a result, augment the heat transfer. Also, *Nu* grows by the *Re* number because of higher velocity which improves boundary layer mixing and improves the convective heat transfer coefficient.

**Figure 9:**
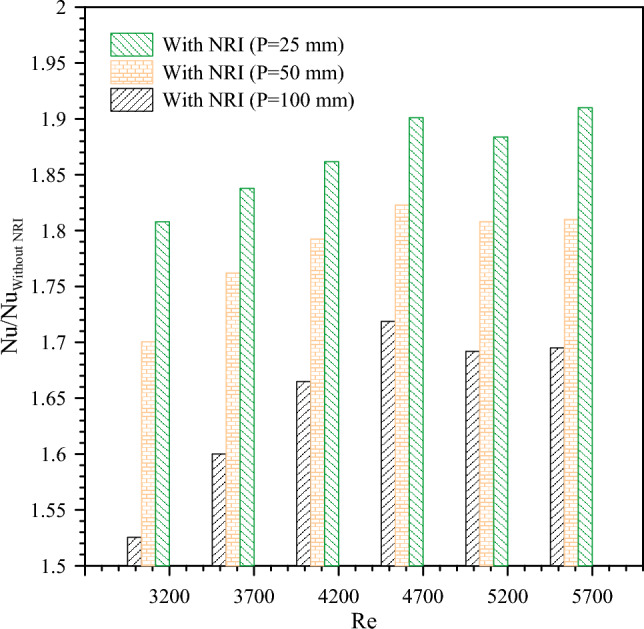
The ratio of *Nu* enhancement versus *Re* for NRI at different pitches (100 mm, 50 mm, and 25 mm).

The *Nu* ratio of the configurations with NRI and the PT is illustrated in Fig. 9 for turbulent flow with three pitch lengths values. the *Nu* ratio increases by pitch decrease for the same values of the *Re* number. That is due to the decrement of the pitch-enhancing swirl flow which interrupts the boundary layers close to the wall causing a growth in heat transfer. Nus for NRI with 25 mm pitch length are 1.81, 1.84, 1.86, and 1.9 times that of the PT at *Re* numbers of 3200, 3700, 4200, and 4700, respectively. It is observed that using NRI has a dominant effect than increasing the *Re* number for the PT.

**Figure 10:**
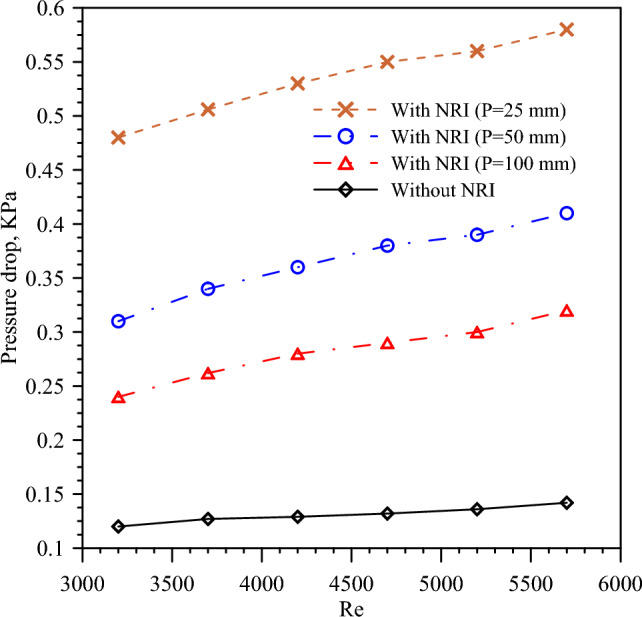
Pressure drop versus *Re* for PT and NRI at different pitches, 100 mm, 50 mm, and 25 mm.

**Figure 11:**
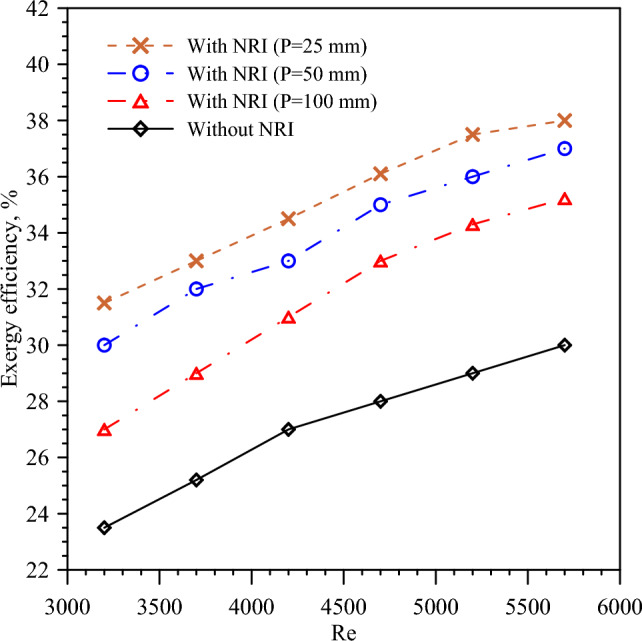
Exergy efficacy versus *Re* for PT and NRI at different pitches (100 mm, 50 mm, and 25 mm).

The Δp is an indication of the required pumping power and is measured as the variance between the inlet and exit pressure of the hot tube. The Δp decreases by nail pitch increment because a high pitch means a small number of barriers which reduces the flow turbulence and vortices. Figure 10 presents the variation of Δp with the *Re* number for different nail pitch values. The figure illustrated that the minimum values of Δp are obtained by PT because there are no barriers that hinder the fluid flow causing swirling. It is found that Δp increases with *Re* number increment that is consistent with Eq. (7) which states that Δp increases by velocity increase. Exergy efficiency is the ratio of the useful work from the system to the reversible work output. Figure 11 presents the exergy efficiency variation with the *Re* number for different nail pitch values and turbulent flow used. Fluid with a high *Re* number and NRI with a small pitch enhances the heat transfer by turbulence and vortices creation of the flow. It is found that exergy efficiency takes the same approach as *Nu* because increasing the heat transfer by nail rod insertion increases *Nu* and exergy efficiency.

### Temperature distribution

**Figure 12:**
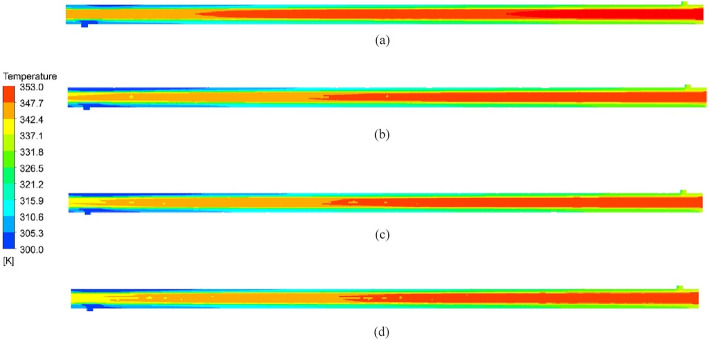
Contours of temperature distribution of DTHX for (**a**) PT, (**b**) NRI at P = 100 mm, (**c**) NRI at P = 50 mm, and (**d**) NRI at P = 25 mm.

The temperature contour in the DTHX is presented in Fig. 12 for an inlet hot water temperature of 353 K. The temperature inside the tube decreases from the right to the left side. The augmentation in the reduction of hot water temperature due to the implementation of NRI stems from the swirling flow it induces, thereby amplifying the degree of tangential contact with the wall, thus facilitating more efficient heat transfer. The highest reduction is obtained with NRI with 25 mm pitch (Fig. 12d) because of the high number of barriers (nails) which increase the turbulence, enhance the flow mixing between the tube core and boundary layers, and augment the heat transfer. It is obvious from (Fig. 12a) that the flow cuts a greater length of the hot PT to reach the same temperature as NRI which proves that NRI enhances the heat transfer.

### Fluid flow structures

**Figure 13:**
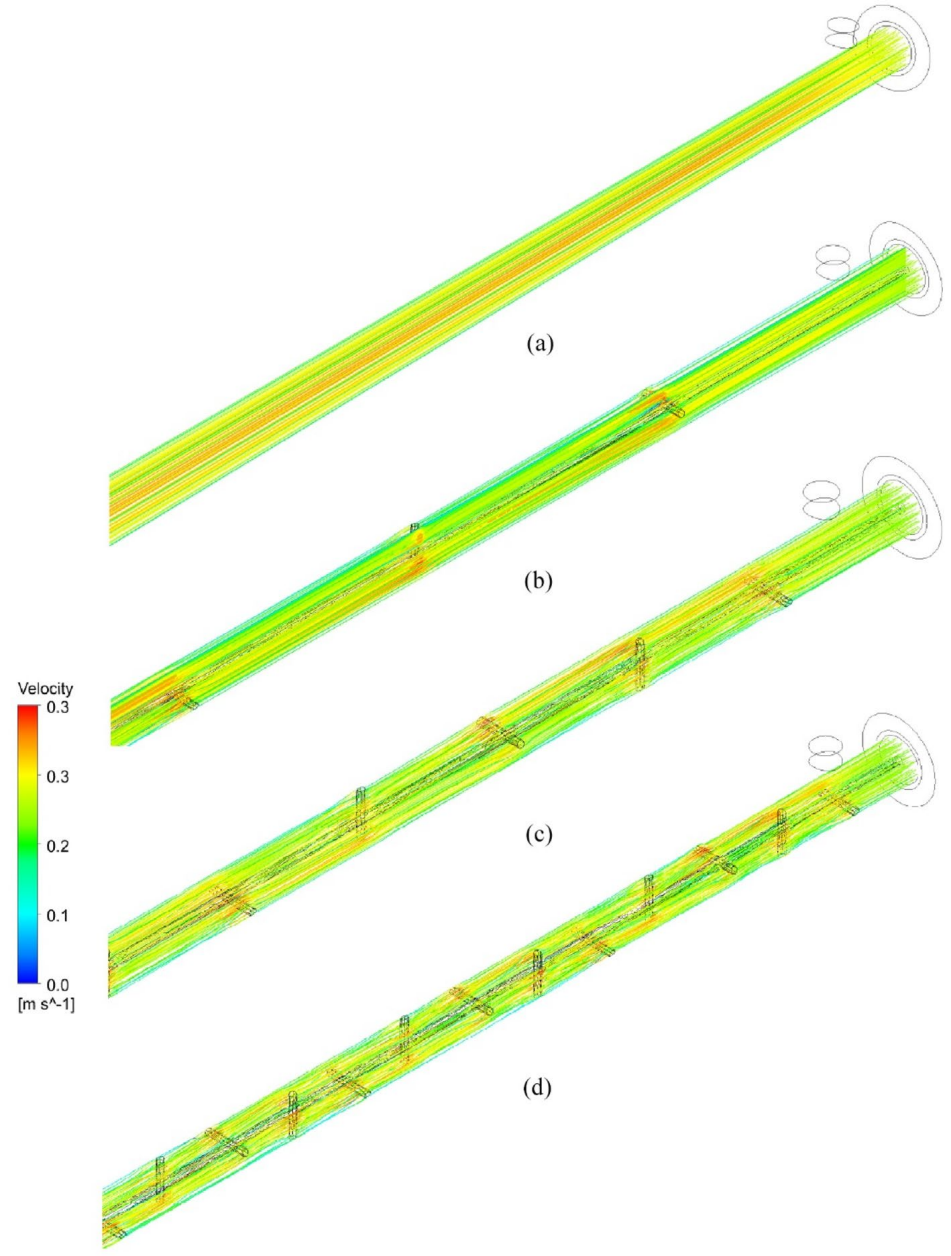
Streamlines of velocity distribution for (**a**) PT, (**b**) NRI at P = 100 mm, (**c**) NRI at P = 50 mm, and (**d**) NRI at P = 25 mm.

**Figure 14:**
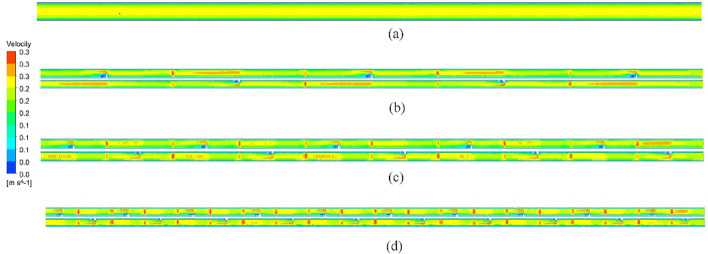
Contours of the velocity distribution of HX tubes for (**a**) PT, (**b**) NRI at P = 100 mm, (**c**) NRI at P = 50 mm, and (**d**) NRI at P = 25 mm.

Streamlines of velocity distribution in the HX for plain and NRI tubes are introduced in Fig. 13. The figure reveals that the velocity is constant along the PT while it increases in the radial direction for the NRI tube. The velocity increases with the pitch length decrease which is due to the disturbance of flow and vortices formation near the NRI surface. Figure 14 illustrates the contours of velocity distribution for PT and NRI configurations. Flow velocity is stable through the PT and increases for the NRI tube. Velocity increases near the rod and this increase becomes higher when the flow confronts the nail's surface. By decreasing the pitch length which means a higher number of nails, the flow velocity investigates the best value. The physical explanation for the previous results is the swirling of flow by rod insertion increases by nail addition which causes vortices near the nails surfaces.

**Figure 15:**
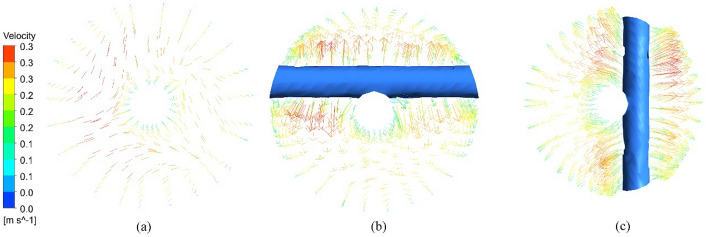
Vectors of the normal velocity for (**a**) PT, (**b**) horizontal nails, (**c**) vertical nails.

**Figure 16:**
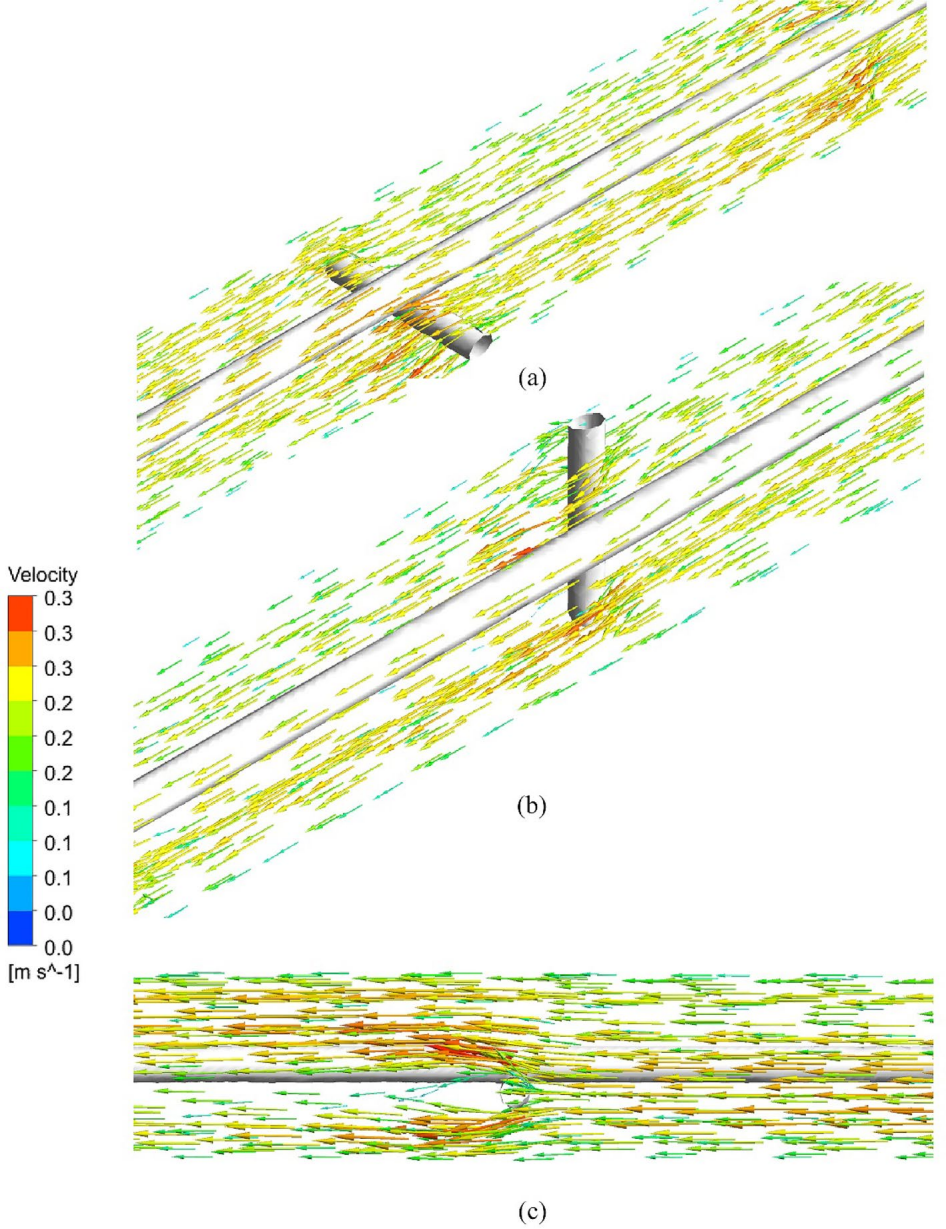
Vectors of velocity distribution for different positions of nails.

Figure 15 shows the vectors of the normal velocity for cross section at PT, horizontal nails, and vertical nails. It is indicated that the horizontal and vertical nails enhance the turbulence and lead to secondary flow that augments the heat transfer compared to plain tube. Figure 16 represents the velocity vector distribution for various positions of nails in a hot tube. The figure shows the velocity of flow past the nails and around the rod. Vector's colors determine the magnitude of the velocity (warmer color shows high velocity). It is illustrated that the fluid moves in a straight direction adjacent to the rod and when it approaches the nail's surface it begins to move over and down the nail's surface increasing its velocity. Also, the velocity in the tube core is higher than that near the tube wall due to the turbulence of flow by NRI.

**Figure 17:**
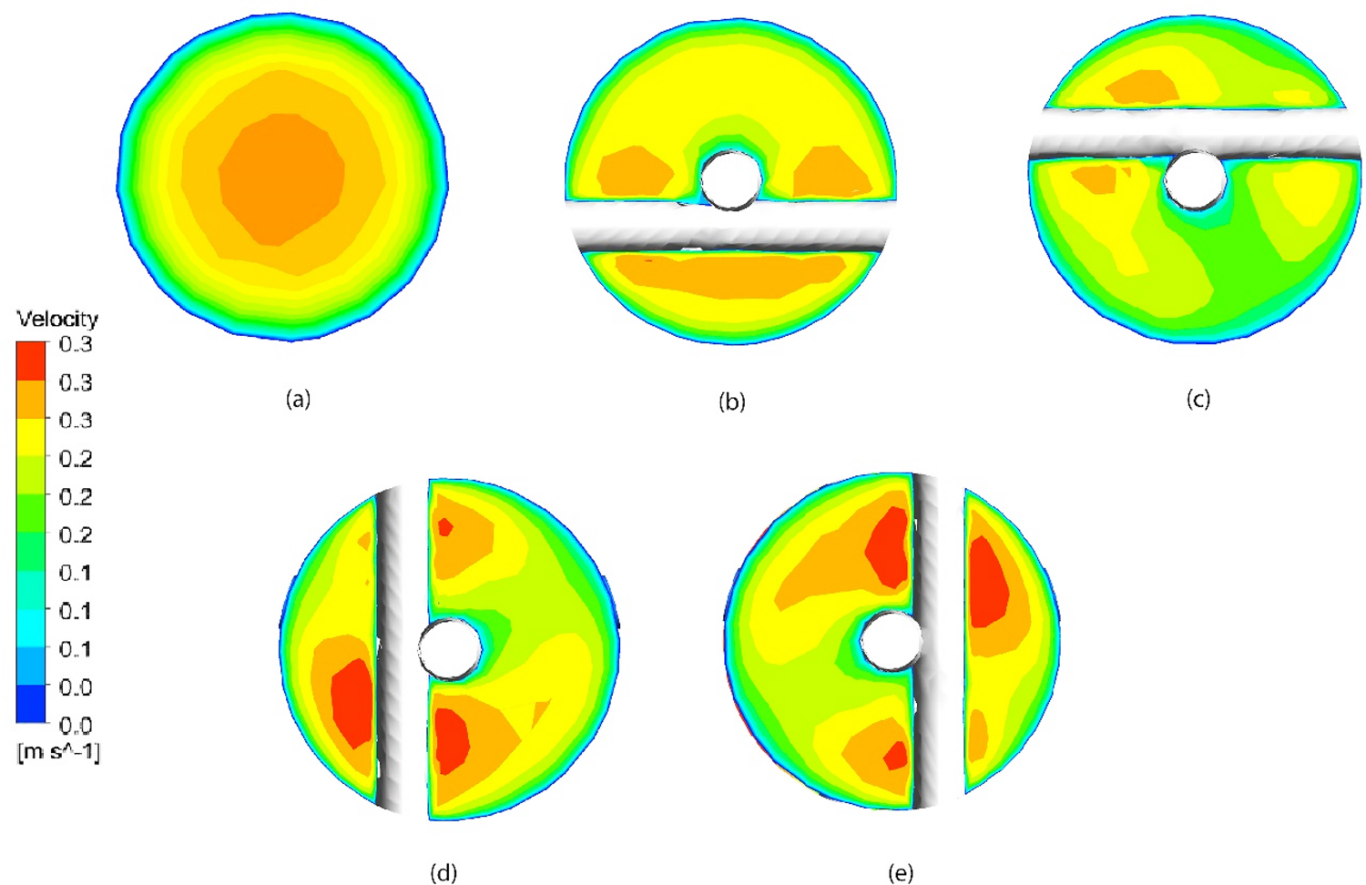
Cross-section view of velocity distribution for (**a**) PT, (**b**) distance = 0.25 m (**c**) distance = 0.75 m (**d**) distance = 0.9 m, and (**e**) distance = 0.5 m.

A cross-section view of velocity distribution in the HX for the PT and for the tube with NRI at different positions along the tube length is represented in Fig. 17. Velocity decreases by radial movement from the tube core to the tube wall in the PT as there is no good mixing of fluid flow. For tubes with NRI, the velocity increases near the turbulator surface, and a better increase occurs by moving through the tube. That is due to colliding with obstacle areas that face the flow causing turbulence and disturbing the boundary layer.

### Pressure distribution

**Figure 18:**
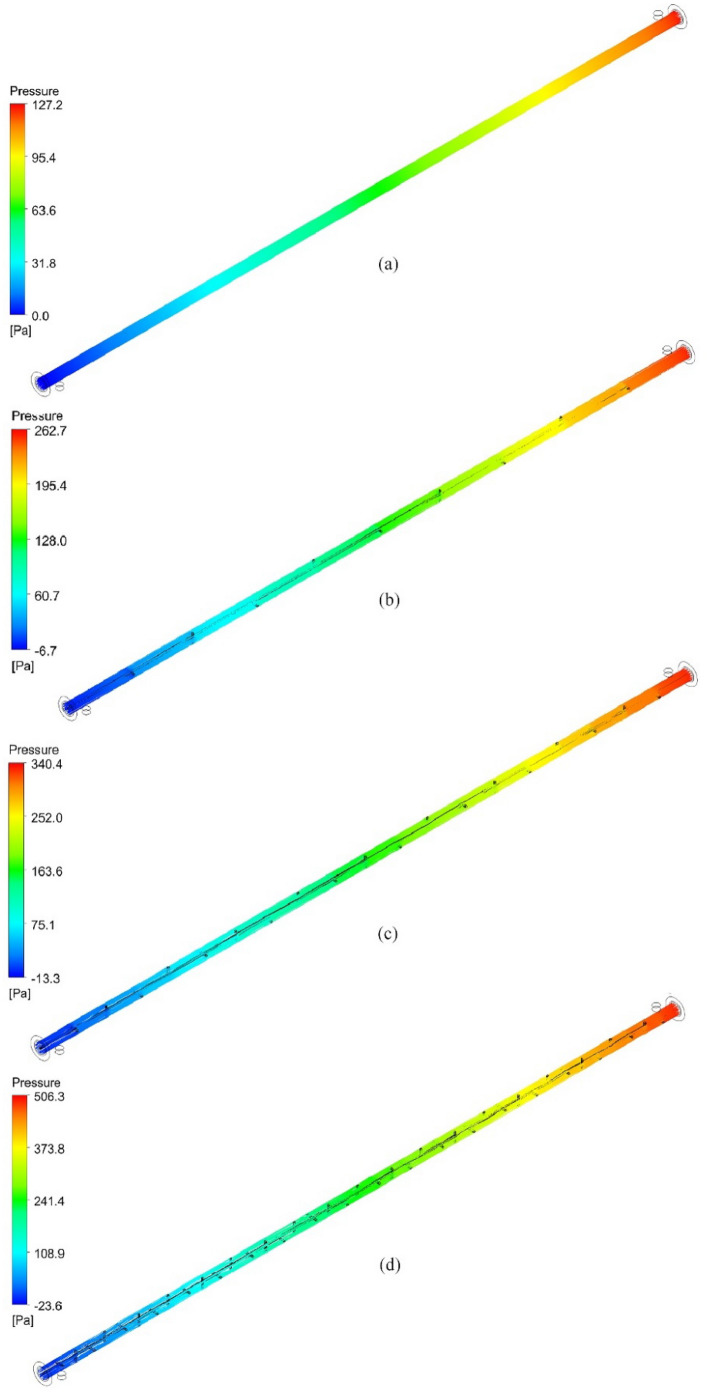
Pressure distribution in the inner tube for (**a**) PT, (**b**) NRI at P = 100 mm, (**c**) NRI at P = 50 mm, and (**d**) NRI at P = 25 mm.

Streamlines of pressure for the hot tube are demonstrated in Fig. 18 for turbulent flow with a *Re* number of 3700. The Δp increases by working fluid flow through the tube for all cases. It is investigated that the Δp increases with higher values by NRI than the PT. That is due to the increase of the area which hinders the flow inside the hot tube and increases the friction coefficient.

## Conclusion

This study investigates the influence of different pitches of the NRI on enhancing heat transfer within DTHX, utilizing both experimental methods and numerical analysis. The NRI offers several advantages over alternative inserts, including its quick manufacturing process, cost-effectiveness, ease of insertion and removal, and reduced fouling propensity. The effects of various NRI pitches, including 100 mm, 50 mm, and 25 mm, on heat transfer performance are studied. Parameters such as the Nu number, exergy efficiency, and Δp are analyzed across a range of Reynolds numbers spanning from 3200 to 5700. The obtained results can be concluded in the following points:

- The insertion of nail rods increases the Nu number where further enhancement can be achieved by reducing the pitch length. The Nusselt number enhancement ratios for NRI with a pitch length of 25 mm are approximately 1.81 to 1.9 times higher than those of the PT.
- Pressure drop increases with the rise of Reynolds numbers for all pitch lengths because of the turbulence and area of the NRI which hinders the flow. The NRI with a 25 mm pitch Length has the maximum pressure drop compared to other pitch Lengths.
- The exergy efficiency increases with NRI for all cases as the Reynolds numbers increase where it increases by 128% for NRI with 25 mm pitch length.
- The Nails rod insert increases the flow turbulence and enhances the heat transfer coefficient where the greatest enhancement is attained at 25 mm pitch length.
- The flow velocity increases near the surfaces of nails due to the disturbance of flow by the insertion and velocity vectors begin to move over and below the nail's surfaces.

## Data Availability

The datasets used and analyzed during the current study are available from the corresponding author upon reasonable request.

## List of symbols

*Δp*: Pressure drop, Pa
*V1*: Water valve
*µ*: Dynamic viscosity, N s/m^2^
*D*_*h*_: Hydraulic diameter, m
*l*: Tube length, m
*P*: Pressure, pa
*v*: Velocity, m/s
*M*: Mass flow rate
*Nu*: Nusselt number
U: Overall heat transfer coefficient, W/m^2^ K
*Re*: Reynolds number
*η*_*ex*_: Exergy efficiency
*f*: Friction factor
$$\rho $$: Density, kg/m^3^
*k*: Thermal conductivity, W/m K

## Subscripts

*in*: Inlet
*out*: Outlet
*as*: Annulus
*int*: Internal
*hw*: Hot water
*cw*: Cold water

## Abbreviations

DTHX: Double tube heat exchanger
NRI: Nails rod insert
TTI: Twisted tape inserts
PT: Plain tube
HTE: Heat transfer enhancement
